# Prenatal diagnosis of micrognathia: a systematic review

**DOI:** 10.3389/fped.2023.1161421

**Published:** 2023-04-12

**Authors:** Zhengqiang Cang, Jiangbo Cui, Jiaomiao Pei, Zheng Wang, Yichen Du, Siqi Mu, Wenjie Dou, Xing Fan, Xi Zhang, Yang Li

**Affiliations:** ^1^ Department of Plastic and Reconstructive Surgery, Xijing Hospital, The Fourth Military Medical University, Xi’an, China; ^2^ Department of Ultrasound Diagnosis, Qinhuang Hospital, Xi’an, China

**Keywords:** micrognathia, fetus, mandible, biometric parameters, prenatal diagnosis

## Abstract

**Purpose:**

This systematic review aimed to analyze the characteristics of different diagnostic techniques for micrognathia, summarize the consistent diagnostic criteria of each technique, and provide a simple and convenient prenatal diagnosis strategy for micrognathia.

**Methods:**

In accordance with the Preferred Reporting Items for Systematic Reviews and Meta-Analyses (PRISMA) guidelines, the search was undertaken in three international databases (PubMed, Scopus, and Web of Science). The three reviewers assessed all papers and extracted the following variables: author's name and year of publication, country, study design, number of participants, gestational age, equipment for prenatal examination, biometric parameters related to micrognathia, main results.

**Results:**

A total of 25 articles included in the analysis. Nineteen articles described cross-sectional studies (76 percent), 4 (16 percent) were case-control studies, and 2 (8 percent) were cohort studies. Fifteen studies (60 percent) had a prospective design, 9 (36 percent) had a retrospective design, and one (4 percent) had both prospective and retrospective design. Thirty-two percent of the studies (*n* = 8) were performed in USA, and the remaining studies were performed in China (*n* = 4), Israel (*n* = 3), Netherlands (*n* = 3), UK (*n* = 1), France (*n* = 1), Italy (*n* = 1), Belgium(*n* = 1), Germany (*n* = 1), Spain (*n* = 1), and Austria (*n* = 1). The prenatal diagnosis of micrognathia can be performed as early as possible in the first trimester, while the second and third trimester of pregnancy were the main prenatal diagnosis period. The articles that were included in the qualitative synthesis describe 30 biometric parameters related to the mandible.

**Conclusion:**

Of the 30 biometric parameters related to the mandible, 15 can obtain the simple and convenient diagnostic criteria or warning value for micrognathia. Based on these diagnostic criteria or warning value, clinicians can quickly make a preliminary judgment on facial deformities, to carry out cytologic examination to further clarify the diagnosis of micrognathia.

## 1. Introduction

Micrognathia is a facial malformation characterized by small mandibular size, giving the fetus the appearance of a small jaw and overbite on profile facial views (1–3). As one of the most common craniofacial deformities, micrognathia is usually accompanied by retrognathia, glossoptosis and obstruction of the upper airways, which may greatly damage infants' appearance, complicate infant feeding, and even bring great risk for infant survival (4–12). Feeding problems is the main cause of poor infant growth and development because infants with upper airway obstruction lack the pulmonary reserve necessary to support the additional respiratory effort required for oral feeding, and have higher caloric consumption caused by repeated attempts to clear their upper airway (9, 12). Respiratory problem may cause perinatal emergency because severe upper airway obstruction may lead to hypoxemia in newborns with micrognathia and glossoptosis (7, 8). Prenatal diagnosis of micrognathia is vital to minimize these risks and avoid unprepared emergencies involving invasive interventions (e.g., tracheostomy) as it facilitates triage for delivery at a tertiary care center equipped with experienced management teams for micrognathia, helps clinicians increase their preparedness and take decisions regarding the management plan in advance, and improves family education.

Prenatal diagnosis of micrognathia was initially mainly determined by observing midsagittal ultrasound imaging of the fetal facial profile without using definitive metrics (13, 14). Prenatal ultrasound screening of the facial profile, length, and sagittal position of the mandible most often depends on subjective standards, usually yielding a low and unsatisfying sensitivity (14). For higher sensitivity and accuracy, various objective techniques have been introduced to help better define micrognathia *in utero*, including the inferior facial angle (IFA), fronto-naso-mental angle (FNMA), mandibular length, jaw index, etc (15–20). However, controversies about simple and efficient methods to evaluate micrognathia still exist, and the diagnostic criterion of specific method for micrognathia lacks consistency in the data. We performed a review of all published studies on prenatal imaging diagnosis of micrognathia to analyze the characteristics of different diagnostic techniques, try to summarize the consistent diagnostic criteria of each technique for micrognathia, and provide a simple and convenient prenatal diagnosis strategy for micrognathia. To the best of our knowledge, this is the first systematic review of prenatal diagnosis of micrognathia.

## 2. Methods

### 2.1 Search strategy

The literature search was undertaken in three international databases: the PubMed, Scopus, and Web of Science until 2022. The Medical Subject Heading (MeSH) terms “micrognathism”, “pierre pobin syndrom”, and “prenatal diagnosis” and all their entry terms were used. A more detailed search strategy for PubMed is available in Supplementary File S1. Search terms and strategy were translated for use with alternative databases.

### 2.2 Eligibility criteria

Three reviewers (ZQC, JBC and JMP) screened title, abstract, and finally full-text articles against inclusion and exclusion criteria, as shown in Table 1.

**Table 1:** Inclusion and exclusion criteria for abstract and full-text screening.

Inclusion criteria
Prospective and retrospective cohort studies, case-control studies, cross-sectional studies, and longitudinal studies on prenatal diagnosis of micrognathia.
Exclusion criteria
Non-imaging Studies
Publication not written in English language
Case reports, reviews, conference abstracts, and letters to editor
Animal studies, cell studies, and cadaver studies

### 2.3 Data extraction and selection

After performing a search in the databases, the necessary data were transferred to the Endnote v9 (Clarivate Analytics, Philadelphia, Pa.) for publishing and managing bibliographies. The process of data extraction was performed based on the Preferred Reporting Items for Systematic Reviews and Meta-Analyses (PRISMA) guidelines. The articles were downloaded from the databases, after which duplicates identified by title and author, were removed. Three reviewers (ZQC, JBC and JMP) screened titles and abstracts of the remaining records. Then, the full-text of relevant articles were read and examined according to the inclusion criteria, to decide whether to include them in the systematic review. Where the three reviewers disagreed, consensus was achieved by discussion with the fourth reviewer (YL). The following information was extracted from each study: author's name and year of publication, country, study design, number of participants, gestational age (GA), equipment for prenatal examination, biometric parameters related to micrognathia, main results. Meta-analysis was not conducted, as the studies were heterogeneous.

## 3. Results

### 3.1 Study selection

**Figure 1:**
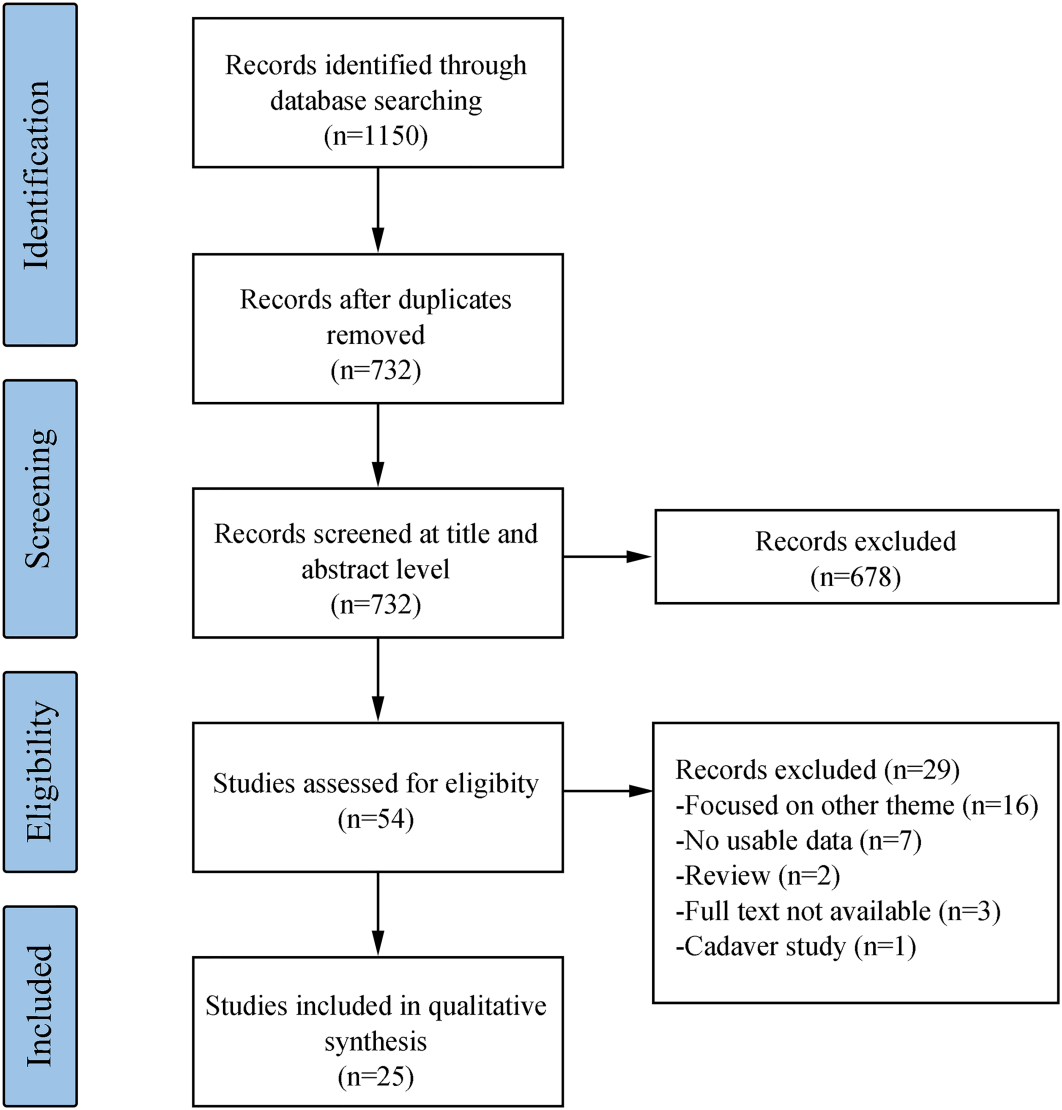
Flowchart of the search strategy and included studies.

Figure 1 shows the flow chart of records selection. A total of 1,150 studies were retrieved through the search queries in all three databases. Following electronic removal of duplicates (*n* = 418), 732 abstracts were screened independently by the three reviewers (ZQC, JBC and JMP) against the eligibility criteria. After excluding 678 abstracts that did not meet the inclusion criteria, 54 full-text articles were further screened by the same two reviewers. After full-text screening, 29 studies were excluded, leaving a total of 25 articles included in the analysis.

### 3.2 Study characteristics

In Table 2, the studies' characteristics are presented. Nineteen articles described cross-sectional studies (76 percent) (15–17, 19–26, 28–33, 36, 40), 4 (16 percent) were case-control studies (34, 35, 37, 39), and 2 (8 percent) were cohort studies (27, 38). Fifteen studies (60 percent) had a prospective design (15–17, 20–28, 30, 31, 36, 40), 9 (36 percent) had a retrospective design (20, 33–35, 37–39), and one (4 percent) had both prospective and retrospective design (32). Thirty-two percent of the studies (*n* = 8) were performed in USA (21–23, 29, 34, 35, 37, 39), and the remaining studies were performed in China (*n* = 4) (20, 25, 36, 40), Israel (*n* = 3) (26, 28, 38), Netherlands (*n* = 3) (27, 30, 31), UK (*n* = 1) (15), France (*n* = 1) (24), Italy (*n* = 1) (16), Belgium(*n* = 1) (17), Germany (*n* = 1) (19), Spain (*n* = 1) (32), and Austria (*n* = 1) (33). From 25 studies included in the qualitative synthesis, 14 studies (56 percent) used two-dimensional (2D) ultrasound for prenatal examination of fetuses (15–17, 19–23, 26, 28, 31, 36, 39, 40), 4 (16 percent) used magnetic resonance imaging (MRI) (33–35, 38), 5 (20 percent) used three-dimensional (3D) ultrasound, 1 (4 percent) used both 2D ultrasound and MRI (37), 1 (4 percent) used both 2D and 3D ultrasound (32). The prenatal diagnosis of micrognathia can be performed as early as possible in the first trimester, while the second and third trimester of pregnancy were the main prenatal diagnosis period. The articles that were included in the qualitative synthesis describe 30 biometric parameters related to the mandible, including IFA, FNMA, maxilla-nasion-mandible angle (MNMA), mandibulomaxillary facia angle (MMFA), frontomaxillary/mandibulomaxillary facial angle (FMFA/MMFA), facial maxillary angle (FMA), mandibular protrusion, maxillary/mandibular protrusion, mandible length, anteroposterior diameter (APD), transverse diameter (TD), APD/TD, jaw index, mandible width (MDW), mandible/maxilla width (MDW/MXW), mandibular body length (MBL), chin index, mandibular corpus length, maxillary/mandibular corpus length, chin length, chin/philtrum length, mandibular vertebral length (MVL), lower-facial depth, mid-facial/lower-facial depth, mandibular curvature, maxillary/mandibular curvature, oropharyngeal space (OPS), fetal profile (FP) line, alveolar overjet (AO), mandibular gap.

**Table 2:** Characteristics of the studies.

Reference	Type of study	Number of participants/ gestation age (weeks)	Diagnostic equipment	Biometric parameters	Main results	
Otto et al. 1991 USA (21)	Prospective cross-sectional study	134/14–39	2D ultrasound	Mandible length	Mandible length = −2.41 + 0.297 GA-0.0027GA^2^	
					Mandible length = −0.198 + 0.565BPD	
					Mandible length = −0.2346 + 0.156HC	
					Mandible length = 0.1399 + 0.781FL-0.0015FL^2^	
Chitty et al. 1993 UK (15)	Prospective cross-sectional study	184/12–28	2D ultrasound	Mandible length	Mandible length = 46.516 + 15.735$\sqrt[{2}]{\mathrm{GA}}$	
					Mandible length = −1.13911 + 0.54018BPD	
					Mandible length = −1.61327 + 0.14423HC	
					Mandible length = 5.4977 + 0.52551 FL + 0.0000314FL^3^	
Watson et al. 1993 USA (22)	Prospective cross-sectional study	204/14–40	2D ultrasound	TD	TD: positive and linear correlation with GA, BPD and FL	
				APD	APD: positive and linear correlation with GA, BPD and FL	
Sivan et al. 1997 USA (23)	Prospective cross-sectional study	200/16–38	2D ultrasound	Chin length	Chin length = −6.5 + 0.7GA	
Paladini et al. 1999 Italy (16)	Prospective cross-sectional study	262/12–37	2D ultrasound	APD	APD: positive and linear correlation with GA and BPD	
				TD	TD: positive and linear correlation with GA and BPD	
				Jaw index	Mean jaw index = 32.2 ± 4.9, 5th Percentiles = 24.0, 95th Percentiles = 41.0	
Rotten et al. 2002 France (24)	Prospective cross-sectional study	371/18–28	3D ultrasound		For variables shown to be independent of GA, the mean and standard deviation (SD) were computed. The mean ± 2 SD interval defined the normal population.	
					IFA: no correlation with GA, mean IFA = 65.5 ± 8.13 (°), IFA < 49.2° defined micrognathia.	
					MD = 7.76 + 0.74GA	
				IFA	MX = 7.41 + 0.75GA	
					MD/MX: no correlation with GA, mean MD/MX = 1.017 ± 0.116, MDW/MXW < 0.785 defined micrognathia.	
				MDW		
				MXW		
				MDW/MXW		
Tsai et al. 2004 Taiwan (25)	Prospective cross-sectional study	183/15–35	3D ultrasound	MBL	MBL: positive and linear correlation with GA	
				Chin index	Chin index: positive and linear correlation with GA	
Gull et al. 2005 Israel (26)	Prospective cross-sectional study	153/13–42	2D ultrasound	Philtrum length	Philtrum length = exp (2.778577-23.476723/GA)	
				Chin length	Chin length = exp (3.7922–28.043/GA)	
				Chin/philtrum length	Chin/philtrum length = 1.7931 + 0.0206GA	
Roelfsema et al. 2006 Netherlands (27)	Prospective, cohort study	126/18–34	3D ultrasound	Maxillary protrusion	Mean maxillary protrusion = 81.08° (80.34° to 81.82°)	
				Mandibular protrusion	Mean mandibular protrusion = 67.25° (66.65° to 67.86°)	
				Maxillary corpus length	Maxillary corpus length = 19.33 + 1.373GA-0.0206GA^2^	
				Mandibular corpus length	Mandibular corpus length =15.58 + 1.154GA + (−0.0102) GA^2^	
				Maxillary/mandibular corpus length	Maxillary/mandibular corpus length = 0.09-0.0019GA	
				Mid-facial depth	Mid-facial depth = 31.22 + 2.011GA-0.0163GA^2^	
				Lower facial depth	Lower facial depth = 28.97 + 1.886GA	
				Mid-facial/lower facial depth	Mid-facial/lower facial depth = 1.08 + 0.0039GA	
				Maxillary curvature	Maxillary curvature = 86.46 + 5.93GA-0.039GA^2^	
				Mandibular curvature	Mandibular curvature = 80.96 + 5.47GA	
				Maxillary/mandibular curvature	Maxillary/mandibular curvature = 1.07-0.0021GA	
Zalel et al. 2006 Israel (28)	Prospective, cross-sectional study	480/11–31	2D ultrasound	TD/APD	TD/APD = 1.7759-0.0105GA (1.3 to 1.5)	
Borenstein et al. 2007 USA (29)	Retrospective, cross-sectional study	200/11–13^+6^	3D ultrasound	FMFA	FMFA = 93.34-0.200CRL (°)	
				MMFA	MMFA = 128.1-0.303CRL (°)	
				FMFA/MMFA	Mean FMFA/MMFA = 0.74 ± 0.07 (SD = 0.070)	
Palit et al. 2008 Belgium (17)	Prospective, cross-sectional study	81/18–35	2D ultrasound	FNMA	Mean FNMA = 146.4 ± 2.77 (°), 5th Percentiles = 142°, 95th Percentiles = 151°	
Jong-Pleij et al. 2011 Netherlands (30)	Prospective cross-sectional study	241/16–36	3D ultrasound	MNMA	Mean = 13.53 (range, 8.96–19.58) (°), 5th Percentiles = 10.39°, 95th Percentiles = 16.91°	
Luedders et al. 2011 Germany (19)	Retrospective, cross-sectional study	54^a^/11^+5^ –35^+6^	2D ultrasound	IFA	Mean IFA = 44.8 (range, 27–49) (°)	
				FNMA	Mean FNMA = 123.3 (range, 100–134) (°)	
Jong-Pleij et al. 2012 Netherlands (31)	Prospective cross-sectional study	237/16–36	2D ultrasound	FP line	Zero: 222 cases (93.7%), positive: 15 cases (6.3%), F distance = 2.8 mm (2.1–3.6 mm)	
Sepulveda et al. 2012 Spain (32)	Prospective and retrospective cross-sectional study	204/11–13	2D and 3D ultrasound	Mandibular gap	Mandibular gap was identified in all 204 normal fetuses, mandibular gap = 0.435 + 0.033CRL	
Nemec et al. 2015 Austria (33)	Retrospective cross-sectional study	355/20–36	MRI	APD	APD = 0.281 + 0.989GA	
				IFA	No correlation with GA, Mean IFA = 67.7 (range, 50.3-86.1) (°), 5th Percentiles = 55.6°, 95th Percentiles = 79.0°	
				Jaw index	Mean jaw index = 39.9 (range, 29.0–52.4), 5th Percentiles = 33.5, 95th Percentiles = 46.8	

Kooiman et al. 2018 USA (34)	Retrospective case-control study	116/25.6 ± 5.1	MRI	PRS group	IFA Jaw index OPS	Mean IFA = 41.2 ± 11.7 (°)
				*n* = 27		Mean jaw index = 35.1 ± 7.7
						Mean OPS = 4.3 ± 2.3
				Micrognathia group *n* = 35		Mean IFA = 53.5 ± 9.3 (°)
						Mean jaw index = 40.5 ± 5.2
						Mean OPS = 5.8 ± 2.0
				Control group		Mean IFA = 59.9 ± 8.6 (°)
				*n* = 54		Mean jaw index = 42.4 ± 4.8
						Mean OPS = 7.1 ± 1.7
Resnick et al. 2018 USA (35)	Retrospective case-control study	162/25.6 ± 4.9	MRI		Three variables were independent predictors of RS: (1) Veau I/II cleft palate (OR = 38.8), (2) TSI (>0.8; OR = 8.7), and (3) IFA(<48°; OR = 14.5).	
Lu et al. 2019 Hong Kong (36)	Prospective cross-sectional study	542/16–31	2D ultrasound	FMA	FMA = 60.3511 + 1.0895GA-0.0004488GA^3^ (°), FMA increased slightly with gestation from 16 weeks till approximately 28–31 weeks, at 1° to 2° per week, and decreased minimally thereafter.	
				FNMA	FNMA = 126.3346 + 0.9529GA-0.0003263GA^3^ (°), FNMA increased slightly with gestation from 16 weeks till approximately 28-31 weeks, at 1° to 2° per week, and decreased minimally thereafter.	
				IFA	IFA = 20.393 + 2.8001GA-0.001033GA^3^ (°), IFA increased slightly with gestation from 16 weeks till approximately 28-31 weeks, at 1° to 2° per week, and decreased minimally thereafter.	
				MNMA	MNMA: no correlation between MNMA and CRL, MNMA = 12.4 with ±2SD of 8.0 and 16.8 (°)	
				FP line	Zero: 513 cases (86.7%), positive: 70 cases (11.8%), negative: 9 cases (1.5%), F distance = 2.9 mm (1.2-5.8 mm)	
Nguyen et al. 2020 USA (37)	Retrospective case-control study	94/24.9 ± 5.2	2D ultrasound and MRI	PRS group *n* = 25	IFA	Ultrasound: Mean IFA = 39.7 ± 7.8 (°)
						MRI: Mean IFA = 41.9 ± 11.8 (°)
				Micrognathia group *n* = 29		Ultrasound: Mean IFA = 48.1 ± 8.6 (°)
						MRI: Mean IFA = 53.6 ± 8.7 (°)
				CLP group *n* = 23		Ultrasound: Mean IFA = 58.8 ± 9.3 (°)
						MRI: Mean IFA = 58.1 ± 10.2 (°)
				Control group *n* = 17		Ultrasound: Mean IFA = 59.3 ± 6.9 (°)
						MRI: Mean IFA = 61.4 ± 9.2 (°)
Toren et al. 2020 Israel(38)	Retrospective cohort study	255/24–36	MRI	IFA	No correlation with GA, mean IFA = 59.597°	
				APD	APD = −0.836 + 1.007GA	
				MVL	MVL = −8.115 + 1.954GA	
Bruce et al. 2021 USA (39)	Retrospective case-control study	96/18–22	2D ultrasound	PRS group *n* = 48	FNMA FMA AO	Mean FNMA = 129.3 ± 8.6 (°)
						Mean FMA = 63.2 ± 9.2 (°)
						Mean AO = 3.9 ± 1.4
				Control group *n* = 48		Mean FNMA = 137.4 ± 3.2 (°)
						Mean FMA = 74.8 ± 6.1 (°)
						Mean AO = 2.1 ± 0.9
Ji et al. 2022 China (40)	Prospective cross-sectional study	800/11–13^+6^	2D ultrasound	IFA	No correlation with CRL, mean IFA = 64.91 ± 6.73 (°), 5th	
					Percentiles = 54°, 95th Percentiles = 79.69°	
				FNMA	FNMA = 163.2–2.94CRL (°)	
				FMFA	FMFA = 92.53–1.71CRL (°)	
				MMFA	MMFA = 118.5–3.75CRL (°)	
				MNMA	MNMA = 9.55 ± 2.84 (°), 5th Percentiles = 4.80°, 95th Percentiles = 14.20°	
					Zero: 177 cases (22.1%), positive: 623 cases (77.9%), negative: 0 cases.	
				FP line	Obvious mandibular gap: 795 cases (99.4%), narrow mandibular gap: 5 cases (0.6%)	

				Mandibular gap		
Li et al. 2022 China (20)	Retrospective cross-sectional study	82/11–13^+6^	2D ultrasound	TD	TD = −15.615 + 1.987GA	
				APD	APD = −8.557 + 1.101GA	
				APD/TD	APD/TD = 0.603–0.003GA	
				Mandible length	Mandible length = 0.861 + 0.137CRL	
				IFA	IFA: positive correlation with CRL, median IFA = 66.5°, 25th percentiles = 62.5°, 75th percentiles = 70.4°	

^a^ 54 fetuses with micrognathia.

GA, gestational age; BPD, biparietal diameter; HC, head circumference; FL, femur length; TD, transverse diameter; APD, anteroposterior diameter; IFA, inferior facial angle; MDW, mandible width; MXW, maxilla width; MBL, mandibular body length; FMFA, frontomaxillary facial angle; MMFA, mandibulomaxillary facia angle; CRL, crown rump length; FNMA, fronto-naso-mental angle; MNMA, maxilla-nasion-mandible angle; FP, fetal profile; PRS, Pierre Robin Sequence; OPS, oropharyngeal space; FMA, facial maxillary angle; CLP, cleft lip and palate; MVL, mandibular vertebral length; AO, alveolar overjet; MRI, magnetic resonance imaging.

## 4. Discussion

This review provides a comprehensive overview of longitudinal changes of mandible-related parameters during fetal development and thereby provides references for prenatal diagnosis of fetal micrognathia. The 30 biometric parameters related to the mandible described in the articles in the qualitative synthesis can be divided into three categories, including angles on facial profile, direct measurements of mandibular biometry, and observational characteristics (Table 3).

**Table 3:** The parameters to assess micrognathia using angles on facial profile, direct measurements of mandibular biometry and observational characteristics.

Biometric parameters	Diagnostic criteria	Warning value
**Angles on facial profile**		
IFA	IFA < 50°	
FNMA	FNMA < 135.91°	
MNMA		MNMA > 14.20°
MMFA and FMFA/MMFA	FMFA/MMFA > 0.75	
FMA	FMA < 66°	FMA < 75°
Mandibular Protrusion and Maxillary/Mandibular Protrusion	Mandibular protrusion < 66.65°	
**Direct measurements of mandibular biometry**		
Mandible Length	Compared with the cross-sectional centile chart	
APD, TD, APD/TD and Jaw Index	APD/TD < 0.45 and jaw index < 24	
MDW and MDW/MXW	MDW/MXW < 0.785	
MBL and Chin Index	Compared with the cross-sectional centile chart	
Mandibular Corpus Length and Maxillary/Mandibular Corpus Length	Maxillary/mandibular corpus length > 0.0558	
Chin Length and Chin/Philtrum Length	Chin/philtrum length < 2.06	
MVL	Compared with the cross-sectional centile chart	
Lower-facial Depth and Mid-facial/Lower-facial Depth	Mid-facial/lower-facial depth > 1.01	
Mandibular Curvature and Maxillary/Mandibular Curvature	Maxillary/mandibular curvature > 1.03	
OPS	—	
**Observational characteristics**		
FP line		Negative FP line
AO	—	
Mandibular Gap		Mandibular gap absence

IFA, inferior facial angle; FNMA, fronto-naso-mental angle; MNMA, maxilla-nasion-mandible angle; MMFA, mandibulomaxillary facia angle; FMFA, frontomaxillary facial angle; FMA, facial maxillary angle; APD, anteroposterior diameter; TD, transverse diameter; MDW, mandible width; MXW, maxilla width; MBL, mandibular body length; MVL, mandibular vertebral length; OPS, oropharyngeal space; FP, fetal profile; AO, alveolar overjet.

### 4.1 Angles on facial profile

#### 4.1.1 IFA

**Figure 2:**
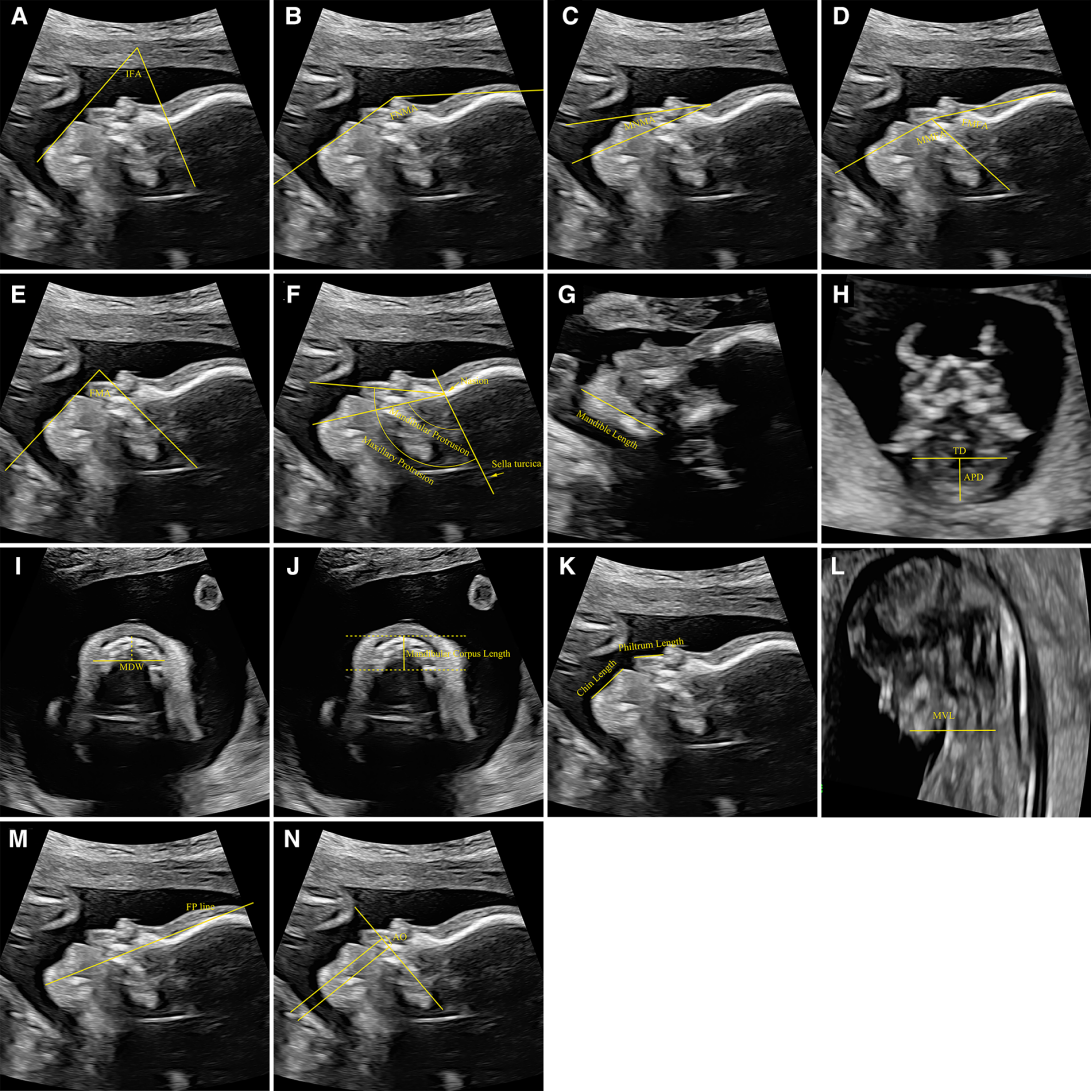
Measurement reference of biometric parameters related to the mandible. (**A**) The measurement of IFA, (**B**) the measurement of FNMA, (**C**) the measurement of MNMA, (**D**) the measurement of MMFA and FMFA, (**E**) the measurement of FMA, (**F**) the measurement of mandibular protrusion and maxillary protrusion, (**G**) the measurement of mandible length, (**H**) the measurement of APD and TD, (**I**) the measurement of MDW, (**J**) the measurement of mandibular corpus length, (**K**) the measurement of chin length and philtrum length, (**L**) the measurement of MVL, (**M**) the measurement of FP line, (**N**) the measurement of AO. IFA, inferior facial angle; FNMA, fronto-naso-mental angle; MNMA, maxilla-nasion-mandible angle; MMFA, mandibulomaxillary facia angle; FMFA, frontomaxillary facial angle; FMA, facial maxillary angle; APD, anteroposterior diameter; TD, transverse diameter; MDW, mandible width; MXW, maxilla width; MVL, mandibular vertebral length; FP, fetal profile; AO, alveolar overjet.

The IFA is measured as the angle between a line vertical to the forehead and a line joining the mentum and protrusive lip (Figure 2A). Of the 25 included studies, 10 measured the IFA of fetuses (19, 20, 24, 33–38, 40). Six of these studies performed the regression analysis between IFA and GA or between IFA and crown rump length (CRL). The study carried out by Li et al. showed a weak positive correlation between IFA and CRL from 11 to 13^+6^ weeks, and the median IFA was 66.5° with 25th and 75th percentiles of 62.5° and 70.4° (20). The study carried out by Lu et al. showed that the IFA increased slightly with gestation from 16 weeks till approximately 28–31 weeks, at 1° to 2° per week, and decreased minimally thereafter (36). Furthermore, in the study of Lu et al., the minimum value of IFA was not less than 60° (36). The other four studies with regression analysis showed that the IFA was independent of GA and CRL, and the IFA of normal fetuses ranged from 50.3° to 86.1°, suggesting that the normal IFA angle can be determined during pregnancy (24, 33, 38, 40). Overall, the IFA range always exceeded 50° throughout pregnancy. In Luedders et al.'s series of 54 prenatal ultrasound studies of fetuses with micrognathia, the average IFA of fetuses with micrognathia was 44.8° (range 27°-49°) (19). The case-control study carried out by Resnick et al. showed the IFA (<48°) is one of the three independent predictors of PRS (35). Therefore, we recommend that IFA < 50° should be used as the diagnostic criteria for micrognathia during prenatal examination.

#### 4.1.2 FNMA

The FNMA is measured as the angle between a line from the most prominent bony part of the forehead to the nasal tip and a line from the most anterior point of the soft tissue of the mandible to the tip of the nose (Figure 2B). Of the 25 included studies, 5 measured the FNMA of fetuses (17, 19, 36, 39, 40). Three of these studies performed the regression analysis between FNMA and GA (or CRL). However, the regression relationships in the three studies are completely different, which may be related to the sample size and trimester difference included in the studies. Based on a small sample size of 81 non-randomly selected fetuses from 18 to 35 gestational weeks, Palit et al. proposed a single cut-off of 142° (5th percentile) across all gestation to define micrognathia (17), but 142° is just around the mean at 19 gestational weeks in the study of Lu et al. (36). The reliability of Palit et al. defined cut-off was also questioned by Luedders et al.'s study, in which the measurements of the FNMA in their cases of micrognathia (range 100°-134°) were far below 142°, and even many normal controls fell below 142° (19). In this review, the lower limit value of the normal FNMA is not less than 135.91°. Therefore, we recommend that FNMA < 135.91° should be used as the diagnostic criteria for micrognathia during prenatal examination.

#### 4.1.3 MNMA

The MNMA is the angle between the lines maxilla-nasion and mandible-nasion in the exact mid-sagittal plane for evaluation of anterior-posterior relationship of the upper and lower jaw. The nasion was defined as the most anterior point at the intersection of the frontal and nasal bones (when there was a little gap between the two of them, the anterior part of the frontal bone at that point was taken) (Figure 2C). Of the 25 included studies, 3 measured the MNMA of fetuses (30, 36, 40). All three studies showed that the MNMA was independent of GA and CRL. Jong-Pleij et al. and Lu et al. proposed that the MNMA can be used in middle and late pregnancy as a useful tool for assessing abnormal facial contours in the fetus, and their results were similar, (mean MNMA was 13.53° and 12.4°, respectively) (30, 36). However, the MNMA measured in the study of Ji et al. from 11 to 13 ^+6^ weeks' gestation was far smaller, with mean only 9.55°±2.84° (5th Percentiles = 4.80°, 95th Percentiles = 14.20°) (40). Such huge gap presumably suggests that it is not easy to measure the MNMA accurately. Therefore, caution should be taken when using the MNMA to assess the micrognathia. Therefore, we recommend that the MNMA > 14.20° should be used as a warning value rather than a diagnostic criteria for micrognathia during prenatal examination.

#### 4.1.4 MMFA and FMFA/MMFA

The upper-anterior corner of the maxilla constitutes the common apex of the frontomaxillary facial angle (FMFA) and MMFA. The first line of the of the FMFA and MMFA coincides and is extended along the upper surface of the palate. The second line of the FMFA is drawn upwards from the anterior aspect of the maxilla at a point where the first line intercepts it. The second arm of the MMFA is drawn downwards and positioned so that the inner aspect of the line was flush with the upper anterior corner of the mandible (Figure 2D). The FMFA provides an objective way of evaluating the position of the maxilla with respect to the fetal forehead, while the MMFA provides an objective method for evaluating the location of the mandible with respect to the maxilla and could be used for early diagnosis of micrognathia. Of the 25 included studies, 2 measured the MMFA and calculated FMFA/MMFA (29, 40). Regression analysis of both studies showed that, in early pregnancy, the MMFA were negatively correlated with CRL while the FMFA/MMFA was independent of CRL. Borenstein et al. reported that the mean MMFA decreased with CRL from 114.5° at a CRL of 45 mm to 103.1° at a CRL of 84 mm (MMFA = 128.1-0.303CRL) with a FMFA/MMFA of 0.74 (29). Ji et al. reported that the mean MMFA ranged from 87° to 101.63° when the CRL was 45–84 mm (MMFA = 118.5–3.75CRL), and the FMFA/MMFA was fixed at 0.75 (40). In the two studies, the normal range of MMFA was wide while the FMFA/MMFA was relatively consistent. It is more reliable and convenient to use the FMFA/MMFA to identify the micrognathia. Therefore, we recommend that the FMFA/MMFA > 0.75 should be used as the diagnostic criteria for micrognathia during prenatal examination.

#### 4.1.5 FMA

The FMA is measured as the angle the line overlying the maxilla and a line across the upper lip and mentum tip (Figure 2E). Of the 25 included studies, 2 measured the FMA of fetuses (36, 39). Lu et al.'s study showed that FMA increased slightly with gestation from 16 weeks till approximately 28–31 weeks, at 1° to 2° per week, and decreased minimally thereafter, and the normal range of FMA was narrow with the lower limit value > 75° (36). Bruce et al.'s study showed that FMA was significantly smaller in the PRS group (63.2 ± 9.2°) compared to the control group (74.8 ± 6.1°), and 52% (*n* = 25) in the PRS group had an abnormally acute FMA (<66°) compared to 2% (*n* = 1) in the control group (39). Therefore, we recommend using FMA < 75° as the warning value and FMA < 66° as the diagnostic criteria for micrognathia during prenatal examination.

#### 4.1.6 Mandibular protrusion and maxillary/mandibular protrusion

The degree of maxillary and mandibular protrusion was determined by the angle between the sella-nasion and nasion-anterior rims of the maxilla and mandible (Figure 2F). Only one study measured the mandibular protrusion (27). There is no correlation between the mandibular protrusion and GA, whereas the maxillary/mandibular protrusion was negatively correlated to GA. The mean mandibular protrusion was 67.25° (66.65° to 67.86°) (27). Therefore, we recommend that mandibular protrusion < 66.65° should be used as the diagnostic criteria for micrognathia during prenatal examination.

### 4.2 Direct measurements of mandibular biometry

#### 4.2.1 Mandible length

The mandible length was measured as the distance between the cartilaginous symphysis menti and the temporomandibular joint (Figure 2G). Of the 25 included studies, 3 measured the mandible length of fetuses (15, 20, 21). In these studies, the mandible length was plotted against GA and CRL as independent variables to build the growth charts, and was positively correlated with GA and CRL in all three trimesters. Considering that the mandible length changes greatly during pregnancy, it is necessary to compare the mandibular length measured during prenatal examination of the fetus with the cross-sectional centile chart for the mandible length if the mandibular length is used to screen micrognathia.

#### 4.2.2 APD, TD, APD/TD and jaw index

The TD of the mandible is measured as the distance from one external bone table to the other when the line between these just touched the anterior portion of the fetal hypopharynx. The APD is measured as the distance from the symphysis mentis to the middle of the TD (Figure 2H). The jaw index was then calculated as follows: APD/biparietal diameter × 100. Of the 25 included studies, 5 measured the APD (16, 20, 22, 33, 38) and 3 measured the TD of fetuses (16, 20, 22). All studies showed both APD and TD were positively correlated with GA. Zalel et al. reported that, in healthy fetuses at 11–31 weeks, there was a positive correlation between APD/TD and GA (28). However, the mean APD/ TD had a small range of 0.66 to 0.76. Li et al. reported that, in healthy fetuses at 11–13^+6^ weeks, the mean APD/TD was 0.56 with 5th and 95th percentiles of 0.45 and 0.75, while, the mean APD/TD in fetuses with micrognathia was <0.42 and significantly lower compared to healthy fetuses (20). The jaw index was calculated in 3 studies. In the study of Paladini et al., the mean jaw index was 32.2 with 5th and 95th percentiles of 24 and 41, and the index allowed a clear separation between micrognathic and normal fetuses (16). Only one fetus with micrognathia had a jaw index greater than 21, and only two fetuses without micrognathia had a jaw index less than 22 (16). The studies carried out by Nemec et al. and Kooiman et al. are consistent with Paladini et al. (33, 34). Considering that APD and TD changes greatly throughout pregnancy, we recommend that APD/TD < 0.45 and jaw index < 24 should be used as the diagnostic criteria for micrognathia during prenatal examination.

#### 4.2.3 MDW and MDW/MXW

The MDW and maxilla width (MXW) were measured on an axial plane caudal to the base of the cranium, at the level of the alveolus (dental arch). A line orthogonal to the sagittal axis was drawn 10 mm posteriorly to the anterior osteous border (approximately at the level of the canines). Measurements were obtained from one external bone table to the other (Figure 2I). The MDW/MXW was derived from these two measurements. Only one study measured the MDW and calculated the MDW/MXW (24). Both MDW and MXW were positively correlated with GA over the 18–28 gestational week period. The MDW/MXW was constant between 18 and 28 gestational weeks, and the mean value was 1.017 ± 0.116. In the study of Rotten et al., the mean ± 2 SD interval defined the normal population. Therefore, we recommend that MDW/MXW < 0.785 should be used as the diagnostic criteria for micrognathia during prenatal examination.

#### 4.2.4 MBL and chin index

The MBL is measured as the distance between the mandibular angles. The ratio of BPD vs. MBL was defined to be the chin index of the fetus. Only one study measured the MBL and calculated the chin index (25). The MBL was positively correlated with GA, whereas the chin index was negatively correlated to GA. In other words, the chin grows wider with the advancement of GA. Consequently, the subjective facial features change from a reverse triangular shape in early weeks, to an oval or square shape in later weeks. Considering that MBL and chin index changes greatly throughout pregnancy, it is necessary to compare the MBL and chin index measured during prenatal examination of the fetus with the cross-sectional centile chart for the MBL and chin index if the MBL and chin index are used to screen micrognathia during prenatal examination.

#### 4.2.5 Mandibular corpus length and maxillary/mandibular corpus length

The mandibular corpus length was represented by the anterior-posterior border of the mandible, which was extended to the frontal rim of the mandibula-gonion (Figure 2J). Only one study measured the mandibular corpus length (27). The mandibular corpus length was positively correlated with GA, whereas the maxillary/mandibular corpus length was negatively correlated to GA. At 18–34 weeks of pregnancy, the mandibular corpus length changed greatly whereas the maxillary/mandibular corpus length had a small range of 0.0254 to 0.0558. Therefore, we recommend that the maxillary/mandibular corpus length > 0.0558 should be used as the diagnostic criteria for micrognathia during prenatal examination.

#### 4.2.6 Chin length and chin/philtrum length

The length of the philtrum was measured from the apex of the philtrum to the lower margin of the upper lip. The length of the chin was measured from the tip of the lower lip to the apex of the chin (Figure 2K). Of the 25 included studies, 2 measured the chin length and 1 calculated the chin/philtrum length (23, 26). Both the chin length and philtrum length were positively correlated with GA. Gull et al. reported the chin/philtrum length as a function of gestational age was relatively constant at 13–42 weeks of pregnancy, and was best described by the linear equation *y* = 0.0206*x* + 1.7931 with a range of 2.06–2.66. Therefore, we recommend that the chin/philtrum length < 2.06 should be used as the diagnostic criteria for micrognathia during prenatal examination.

#### 4.2.7 MVL

The MVL is measured as the distance between the mentum and the anterior vertebral line (Figure 2L). Only one study measured the MVL (38). The MVL was positively correlated with GA and had a wide range. It is necessary to compare the MVL measured during prenatal examination with the cross-sectional centile chart for the MVL if the MVL is used to screen micrognathia.

#### 4.2.8 Lower-facial depth and mid-facial/lower-facial depth

The mid-facial and lower facial depths were determined by the tragus-anterior rim of the maxilla and tragus-gnathion. Only one study measured the lower-facial depth (27).

Throughout pregnancy, the lower facial depths were positively correlated with GA and had a wide range, whereas the mid-facial/lower-facial depth was negatively correlated to GA and relatively constant with a range of 0.95–1.01. Therefore, we recommend that the mid-facial/lower-facial depth > 1.01 should be used as the diagnostic criteria for micrognathia during prenatal examination.

#### 4.2.9 Mandibular curvature and maxillary/mandibular curvature

The maxillary and mandibular curvature was represented by the curvature from the tragus-anterior rim of the maxilla, multiplied by two, and the curvature from the tragus-gnathion, multiplied by two. Only one study measured the mandibular curvature (27). Throughout pregnancy, the mandibular curvature was positively correlated with GA and changed greatly, whereas the maxillary/mandibular curvature was negatively correlated to GA and relatively constant with a range of 1.00–1.03. Therefore, we recommend that the maxillary/mandibular curvature > 1.03 should be used as the diagnostic criteria for micrognathia during prenatal examination.

#### 4.2.10 OPS

The OPS was measured as the distance between the anterior and posterior walls of the oropharynx along a line connecting soft tissue overlying most concave point of mandibular symphysis mentis) and the tongue base located at the intersection of the base of tongue and epiglottis. In the study of Kooiman et al., the OPS was significantly smaller in the RPS group (4.3 ± 2.3 mm) than in the micrognathia group (5.8 ± 2.0 mm) and control group (7.1 ± 1.7 mm) (34). The OPS was also significantly smaller in the micrognathia group than in the control group. However, there is no cross-sectional study on the changes of OPS during pregnancy.

### 4.3 Observational characteristics

#### 4.3.1 FP line

The FP line is a line that passes through the midpoint of the anterior border of the mandible and the nasion (Figure 2M). When the FP line passes lengthwise through the frontal bone, its position is defined as “zero”; when FP line passes the frontal bone anteriorly, its position is defined as negative; when the FP line passes the frontal bone posteriorly, its position is defined as positive. Of the 25 included studies, 3 measured the FP line (31, 36, 40). Jong-Pleij et al. reported that most seen was an FP line with position zero, and the positive FP line never occurred before 27 weeks’ gestation at 16–36 weeks of pregnancy (31). According to study carried out by Lu et al., the prevalence of the negative FP line decreased from 7.1% to 0% by 24 weeks and the positive type was observed from 22 weeks (36). The transition from the negative type to the positive type may result in a false positive result at early second trimester. For all that, the negative FP line should still be regarded as a warning value for micrognathia during prenatal examination.

#### 4.3.2 AO

To determine the AO, the maxillary plane was used as an easy to identify landmark. Two lines perpendicular to the maxilla were then created; the first aligned with the most anterior portion of the maxillary alveolar process (1), and the second aligned with the most anterior portion of the mandibular alveolar process (2). The distance between (1) and (2) was then measured as the alveolar overjet (AO) (Figure 2N). Only one study observed the AO (39). In the study of Bruce et al., the PRS group demonstrated significantly larger AO compared to the control group, 3.9 ± 1.4 mm and 2.1 ± 0.9 mm, respectively. However, there is no cross-sectional study on the AO during pregnancy.

#### 4.3.3 Mandibular gap

The mandibular gap was measured from the midpoint of the echogenic edge of one mandibular bone to the other. Two study observed the mandibular gap (32, 40). In the study of Sepulveda et al., the mandibular gap was identified in all normal fetuses (32). In the study of Ji et al. 99.4% fetuses were observed the presence of the obvious mandibular gap, and 0.6% fetuses were observed the presence of the narrow mandibular gap (40). The absence of the mandibular gap should be regarded as a warning value for micrognathia during prenatal examination.

Most of the facial profile angles are independent of GA, or have a weak positive correlation with GA with a small changing amplitude throughout pregnancy. By comparing the value of these facial profile angles with corresponding diagnostic criterion or warning value in our review, they can be directly used as a reference for the diagnosis of micrognathia. In this review, a total of 5 facial profile angles can be directly used for the prenatal diagnosis of micrognathia, including IFA < 50°, FNMA < 135.91°, MNMA > 14.20°, FMA < 66°, Mandibular protrusion < 66.65°, while only one facial profile angle (MMFA) needs to be calculated as a ratio (FMFA/MMFA) to evaluate indirectly the mandibular abnormality, because the normal range of MMFA is wide and the FMFA/MMFA is relatively consistent. Direct measurements of mandibular biometry usually have a significant correlation with GA with a big changing amplitude throughout pregnancy. They are unavailable for the diagnosis of micrognathia unless compared with the cross-sectional centile chart, or calculated as a ratio such as APD/TD < 0.45, jaw index < 24, MDW/MXW < 0.785, maxillary/mandibular corpus length > 0.0558, chin/philtrum length < 2.06, mid-facial/lower-facial depth > 1.01, maxillary/mandibular curvature > 1.03. Observational characteristics can quickly indicate the presence of micrognathia, and are the most concise biometric parameters. There are two observational characteristics that can be used as warning value for micrognathia, such as the negative FP line and mandibular gap absence. Therefore, we recommend the observational characteristics and facial profile angles as the main diagnostic reference and direct measurements of mandibular biometry as the secondary diagnostic reference for the prenatal diagnosis of micrognathia.

In the included studies, only one evaluated the association between IFA measurements on ultrasound and MRI (37). There is a moderate positive correlation between IFA measurements on ultrasound and MRI. Nguyen et al. believed that a postnatal diagnosis of Robin sequence whose defining features is the micrognathia may be predictable by prenatal MRI (37). MR imaging has shown superiority over conventional sonographic methods in assessing fetal facial structures, as it is of high diagnostic value in cases of limited acoustic window (e.g., maternal obesity, oligohydramnios, and anterior spine position) (41). MRI, however, is a limited resource and requires recognition of risk factors to indicate referral. Ultrasound is a simple measurement equipment that indicates a high risk of micrognathia and prompts a confirmatory MRI study which may improve the availability and reliability of prenatal diagnosis of micrognathia.

In this review, four studies are from China, and the rest are from countries with Caucasian ethnic distribution. However, the limited sample size cannot be used to analyze the impact of ethnic groups on biometric parameters related to the mandible. Further research may be required to clarify the impact of ethnic groups on biometric parameters related to the mandible.

## 5. Conclusions

In conclusion, 30 biometric parameters related to the mandible measured from ultrasound or MRI images have been proposed for screening micrognathia. Among these parameters, 15 can obtain the simple and convenient diagnostic criteria or warning value for micrognathia, including the IFA < 50°, FNMA < 135.91°, MNMA > 14.20°, FMFA/MMFA > 0.75, FMA < 66°, mandibular protrusion < 66.65°, APD/TD < 0.45, jaw index < 24, MDW/MXW < 0.785, maxillary/mandibular corpus length > 0.0558, chin/philtrum length < 2.06, mid-facial/lower-facial depth > 1.01, maxillary/mandibular curvature > 1.03, negative FP line, mandibular gap absence. Based on these diagnostic criteria or warning value, clinicians can quickly make a preliminary judgment on facial deformities, to carry out cytologic examination to further clarify the diagnosis of micrognathia.

## Author contributions

YL, XZ and XF conceived and designed the study. ZC, JC and JP conducted the literature search. ZC, ZW, YD, SM, and WD performed data extraction. ZC, YL and XZ conducted the literature quality assessment. ZC, JC and JP drafted the manuscript with the help of all the other authors. YL and XF reviewed and revised the manuscript. All authors contributed to the article and approved the submitted version.
