# Supplementary material for: Prenatal diagnosis of micrognathia: a systematic review
Source: Front Pediatr. 2023 Apr 12;11:1161421. doi: 10.3389/fped.2023.1161421 (PMC10130438; doi:10.3389/fped.2023.1161421)
Supplement: Supplementary file 1 [file Table1.pdf]

## *Supplementary Material*

# Prenatal Diagnosis of Micrognathia: A Systematic Review

Zhengqiang Cang[1†], Jiangbo Cui[1†], Jiaomiao Pei[1†], Zheng Wang[2], Yichen Du[1], Siqi Mu[1], Wenjie Dou[1], Xing Fan[1]*, Xi Zhang[1]*, Yang Li[1]*

[1]Department of Plastic and Reconstructive Surgery, Xijing Hospital, The Fourth Military Medical University, Xi'an, China

[2]Department of Ultrasound Diagnosis, Qinhuang Hospital, Xi'an, China

***CORRESPONDENCE:**

Yang Li

liyangzx@fmmu.edu.cn

Xi Zhang

jessii@163.com

Xing Fan

fanxing.612@163.com

[†]These authors have contributed equally to this work

**Supplementary Table**

**SUPPLEMENTARY TABLE 1. search strategy for PubMed**

| |
|---|
| Date |
| 28/11/2022 |
| Search database |
| PubMed |
| Search keyword |
| ((("Micrognathism"[Mesh]) OR ((((((((((((((((Micrognathisms[Title/Abstract]) OR (Mandibular Micrognathism[Title/Abstract])) OR (Mandibular |

Micrognathisms[Title/Abstract])) OR (Micrognathism, Mandibular[Title/Abstract])) OR (Micrognathisms, Mandibular[Title/Abstract])) OR (Micrognathia[Title/Abstract])) OR (Micrognathias[Title/Abstract])) OR (Congenital Micrognathism[Title/Abstract])) OR (Congenital Micrognathisms[Title/Abstract])) OR (Micrognathism, Congenital[Title/Abstract])) OR (Micrognathisms, Congenital[Title/Abstract])) OR (Congenital Micrognathia[Title/Abstract])) OR (Congenital Micrognathias[Title/Abstract])) OR (Micrognathia, Congenital[Title/Abstract])) OR (Micrognathias, Congenital[Title/Abstract])) OR (Mandibular Micrognathia[Title/Abstract])) OR (Mandibular Micrognathias[Title/Abstract])) OR (Micrognathia, Mandibular[Title/Abstract])) OR (Micrognathias, Mandibular[Title/Abstract]))) OR (("Pierre Robin Syndrome"[Mesh]) OR ((((((((((((Robin Syndrome, Pierre[Title/Abstract]) OR (Syndrome, Pierre Robin[Title/Abstract])) OR (Robin Sequence[Title/Abstract])) OR (Sequence, Robin[Title/Abstract])) OR (Pierre Robin's Sequence[Title/Abstract])) OR (Pierre Robins Sequence[Title/Abstract])) OR (Sequence, Pierre Robin's[Title/Abstract])) OR (Pierre-Robin Syndrome[Title/Abstract])) OR (Syndrome, Pierre-Robin[Title/Abstract])) OR (Glossoptosis, Micrognathia,[Title/Abstract] AND Cleft Palate[Title/Abstract])) OR (Pierre Robin Sequence[Title/Abstract])) OR (Sequence, Pierre Robin[Title/Abstract])))) AND (("Prenatal Diagnosis"[Mesh]) OR (((((((((((((((((((((((Prenatal Diagnoses[Title/Abstract]) OR (Intrauterine Diagnosis[Title/Abstract])) OR (Diagnosis, Intrauterine[Title/Abstract])) OR (Intrauterine Diagnoses[Title/Abstract])) OR (Diagnosis, Prenatal[Title/Abstract])) OR (Antenatal Diagnosis[Title/Abstract])) OR (Antenatal Diagnoses[Title/Abstract])) OR (Diagnosis, Antenatal[Title/Abstract])) OR (Fetal Screening[Title/Abstract])) OR (Fetal Screenings[Title/Abstract])) OR (Screening, Fetal[Title/Abstract])) OR (Prenatal Screening[Title/Abstract])) OR (Prenatal Screenings[Title/Abstract])) OR (Screening, Prenatal[Title/Abstract])) OR (Antenatal Screening[Title/Abstract])) OR (Antenatal Screenings[Title/Abstract])) OR (Screening, Antenatal[Title/Abstract])) OR (Fetal Diagnosis[Title/Abstract])) OR (Diagnosis, Fetal[Title/Abstract])) OR (Fetal Diagnoses[Title/Abstract])) OR (Fetal Imaging[Title/Abstract])) OR (Fetal Imagings[Title/Abstract])) OR (Imaging, Fetal[Title/Abstract])))
